# Disappearing “Myxoma”: Left Atrial Thrombus Masquerading as a Myxoma

**DOI:** 10.7759/cureus.8536

**Published:** 2020-06-09

**Authors:** Daniel Antwi-Amoabeng, Charles E Willyard, Nageshwara Gullapalli, Michael Keogh

**Affiliations:** 1 Internal Medicine, University of Nevada Reno School of Medicine, Reno, USA; 2 Cardiology, VA Sierra Nevada Health Care System, Reno, USA

**Keywords:** intracardiac mass, intracardiac thrombus, atrial myxoma, echocardiography

## Abstract

Intracardiac masses can be challenging to differentiate by echocardiography. We present a case of several intracardiac masses with echocardiographic features of both thrombi and myxoma in a patient with heart failure symptoms. The masses were confirmed to be thrombi after complete resolution on repeat echocardiography following anticoagulation. Echocardiography complements the history and physical exams in diagnosing intracardiac masses but may present a diagnostic challenge when features are not pathognomonic. Follow up imaging after anticoagulation should be standard of care to avoid unnecessary surgeries when the diagnosis of a cardiac mass is uncertain.

## Introduction

Cardiac myxomas are the most common primary cardiac tumors [1]. They account for approximately 75% of all primary cardiac tumors, they are benign, and are often present in the third to sixth decade of life [1-2]. Depending on the size and location of the tumor, cardiac myxomas may present with no specific physical exam manifestations, or with nonspecific heart failure symptoms including dyspnea, fatigue, or orthopnea. Left atrial thrombi are far more common and are present in 13% of patients with atrial fibrillation who are not anticoagulated [3]. Echocardiography can be helpful in distinguishing the type of cardiac mass. Surgical resection is the treatment of choice for cardiac myxomas and anticoagulation is generally recommended for the initial treatment of intracardiac thrombi [4-5]. Delay in surgery for cardiac myxomas may lead to embolization or further growth and obstruction of surrounding structures leading to worsening symptoms [6].

## Case presentation

A 74-year-old Caucasian male presented to the ED with two months’ history of progressively worsening shortness of breath with exertion and lower extremity edema. The patient also complained of worsening orthopnea over the past several days. In the ED, the patient’s blood pressure was 152/95 mmHg, and his pulse was 95. He was afebrile. He was breathing on room air with a 95% oxygen saturation. The cardiovascular exam revealed a regular rate and rhythm. There was a third heart sound. There were no murmurs. The patient also exhibited 1+ pedal edema in both of his lower extremities. The patient had a medical history significant for nonobstructing three-vessel coronary artery disease diagnosed by cardiac catheterization five years prior, dilated cardiomyopathy, heart failure with reduced ejection fraction (20%-25%) based on echocardiogram five years prior, hypertension, hyperlipidemia, peripheral vascular disease, alcohol use disorder for 10 years, and chronic obstructive pulmonary disease (COPD). The patient had a 60 pack-year smoking history and drank one to two bottles of liquor per day. His medications were aspirin, carvedilol, lisinopril, and atorvastatin. He was noncompliant with his medications and follow ups for chronic medical conditions.

**Figure 1:**
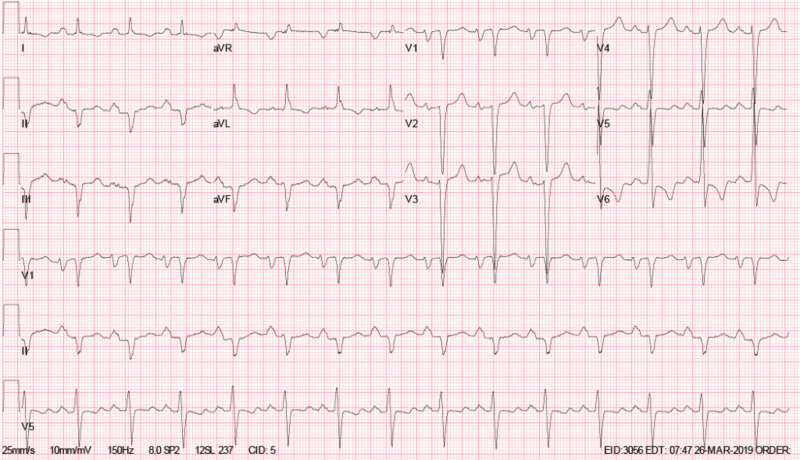
Electrocardiogram: rate 88, normal sinus rhythm, bi-atrial enlargement, left axis deviation, intra-ventricular conduction delay (QRS duration 128 msec), inferior infarct, age undetermined, T-wave inversion in the lateral leads.

**Figure 2:**
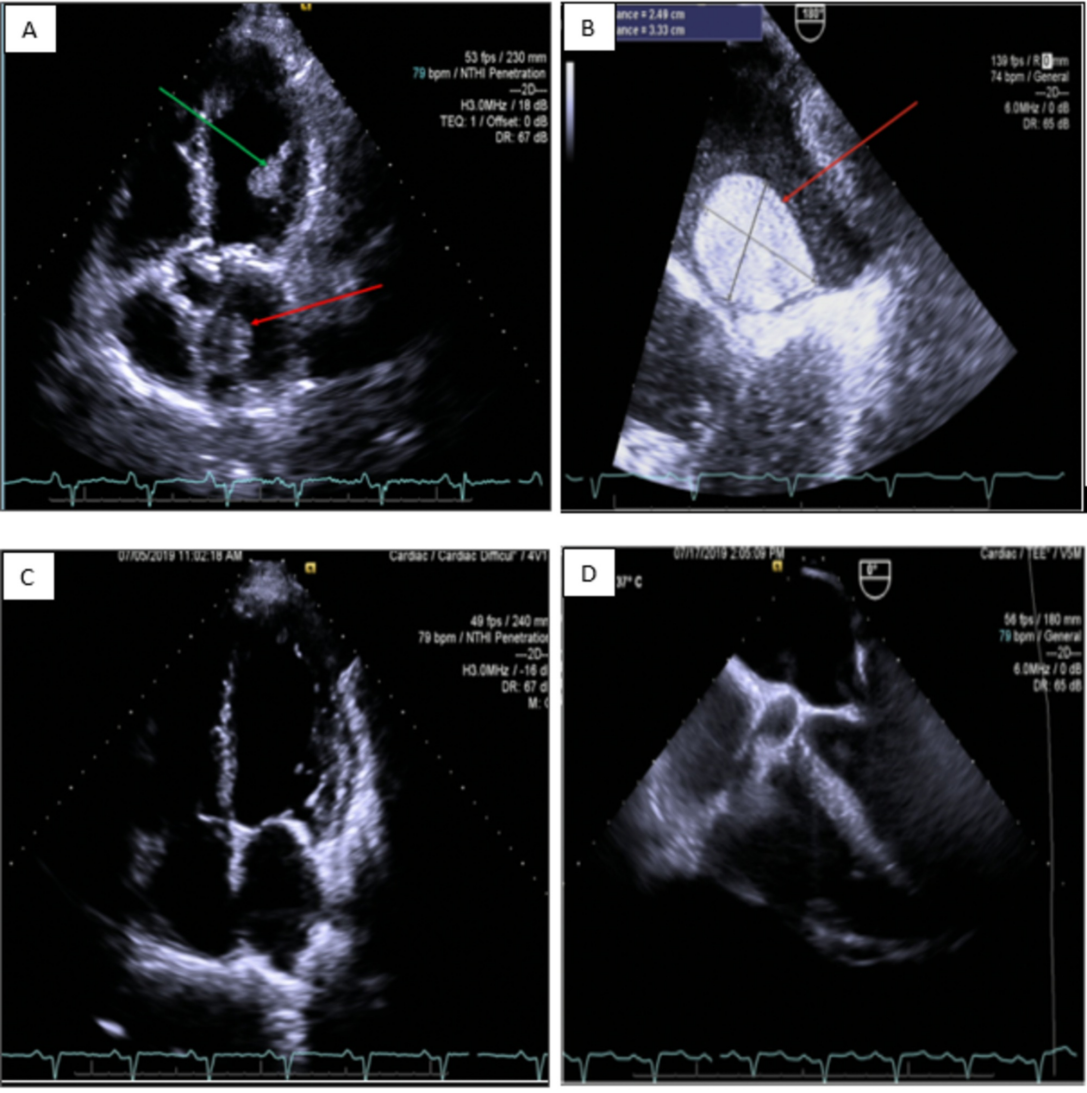
A: Pre-anticoagulation TTE. LA mass suggestive of myxoma (red arrow) as well as a LV thrombus (green arrow). B. Pre-anticoagulation TEE: 2.49 cm x 3.33 cm mass (red arrow) suggestive of myxoma on anteromedial aspect of LA. C: Post-warfarin TTE three months later. Thrombi in the LV and LA have resolved. D. Post-warfarin TEE three months later. No evidence of myxoma. TTE: transthoracic echocardiogram; TEE: transesophageal echocardiogram; LA: left atrium; LV: left ventricle.

On presentation, laboratory findings were significant for a B-type natriuretic peptide >5000 pg/mL. Electrocardiogram showed normal sinus rhythm, bi-atrial enlargement, left axis deviation, intra-ventricular conduction delay (QRS duration 128 msec), inferior infarct, age undetermined, and T-wave inversion in the lateral leads (Figure 1). He received IV furosemide; fluid intake was restricted to 1.5 L daily with a low-salt diet while in the hospital. Transthoracic echocardiogram (TTE) showed a severely dilated left ventricle (LV) with severe global hypokinesis and left ventricular ejection fraction (LVEF) of 10%-20% with a moderately dilated left atrium and a left atrial mass suggestive of a myxoma in addition to thrombi in the LV (Figure 2A). A transesophageal echocardiogram (TEE) performed a day later to better evaluate the left atrial mass revealed a 2.49 cm x 3.33 cm left atrial mass suggestive of a myxoma on the anteromedial aspect of the left atrium, just superior to the membrane of the fossa ovalis (Figure 2B). There was spontaneous echo contrast (SEC). The patient was in normal sinus rhythm during these studies. 

 

As the patient had improved symptomatically, he was discharged home on the fifth day of hospitalization with a three-month course of warfarin for treatment of the LV thrombus. He was started on a therapeutic dose of enoxaparin while inpatient and bridged to warfarin on discharge. Plans were made for a repeat TEE in order to confirm the resolution of the thrombus prior to surgery for the presumed atrial myxoma. The patient’s INR was followed in the outpatient warfarin clinic. Mean INR was 2.2, and ranged from 1.2 to 5.0, with the median being 1.1. INR was within the target 2-3, 46% of the time, and below the target 46% of the time. There was a single reported INR above 3, representing 8% of the readings.

After three months of anticoagulation, repeat TTE revealed that in addition to the LV thrombus, the left atrial mass was no longer present (Figure 2C). There was a modest improvement in ejection fraction (EF) to 20%-25%. TTE also showed decreased aortic valve excursion consistent with a low-flow state. The patient reported compliance with warfarin, carvedilol, atorvastatin, and lisinopril. TEE two days later confirmed a complete resolution of atrial “myxoma” (Figure 2D). At this point, it was concluded that the patient’s presumed atrial myxoma was in fact a thrombus that had resolved with warfarin treatment. Plans for surgery were canceled. Due to his ongoing alcoholism with increased risk of falls, and noncompliance, we elected to discontinue his warfarin. The patient was instructed to continue aspirin, carvedilol, lisinopril, atorvastatin, and furosemide and to follow up with cardiology for ongoing management of heart failure.

## Discussion

This case illustrates the diagnostic challenge often encountered when evaluating patients with intracardiac masses. This difficulty was further complicated by echocardiography findings which supported the diagnosis of both atrial myxoma and thrombus. The patient had history and findings suggestive of both atrial myxoma and thrombi. His dyspnea and fatigue could be explained by either a myxoma or worsening dilated cardiomyopathy, which would represent a low flow state, making the probability of intracardiac thrombi more likely. The patient had SEC on TEE. SEC occurs more frequently in patients with chronic atrial fibrillation and thromboembolic events [7].

Several factors made a left atrial thrombus questionable. The patient was in normal sinus rhythm throughout his hospitalization; he did not have a history of atrial tachyarrhythmias and did not have stenotic atrioventricular valves [8]. Myxomas are the most common nonmalignant cardiac tumors [1]. We considered left atrial myxoma as an alternate diagnosis because of the globular and pedunculated appearance of the mass on the anteromedial aspect of the left atrium. Fatigue and dyspnea with or without palpitations although nonspecific, are the most reported presenting symptoms in patients with myxoma [1]. Our patient presented with a three-month history of dyspnea and fatigue, which was worse than his baseline symptoms in the setting of treated COPD and heart failure.

The patient’s symptoms were nonspecific and echocardiography findings were not discriminatory. On echocardiography, both myxomas and thrombi appear heterogeneous [9]. Thrombi are often multiple, are of various sizes, and occur in varied locations. The left atrium is a common location in those with atrial fibrillation. Myxomas are usually solitary, but multiple myxomas have been reported [10]. They may be pedunculated or sessile and may exhibit intralesional hemorrhage, degeneration, or calcification, which can present as central hypo or hyperechogenicity, and may help identify myxomas [1, 11-12]. However, chronic thrombi can exhibit these same features. The left atrial mass had a stalk and had central hypoechogenicity (Figure 2A). Some authors consider the stalk as a distinguishing feature of myxoma [11, 13].

Two features favored a diagnosis of atrial thrombus over myxoma in this case. First, there was a SEC, which is strongly associated with atrial fibrillation and thromboembolic events [7]. SEC while in normal sinus rhythm is an interesting finding but it made a consideration of atrial myxoma a reasonable speculation. Second, the anteromedial attachment of the presumed atrial myxoma was an unusual location as most atrial myxoma arises from the interatrial septum [14].

There have been reported cases of concurrent left atrial myxoma and thrombi, which required surgical excision to confirm the diagnosis [12-13]. The presence of intracardiac myxoma may present with symptoms of low flow state, which can predispose patients to thrombi formation [13, 15]. We could not definitively exclude atrial myxoma in our patient. Therefore, we planned for surgical excision of the left atrial mass and recommended anticoagulation for the LV thrombus while waiting for improvement in LV function with augmented heart failure treatments as he was considered a high-risk surgical candidate. We elected to perform follow up TEE before surgery because of uncertainty regarding the identity of the left atrial mass. This was the right decision as the echo showed complete resolution of the left atrial mass and we avoided an unnecessary high-risk surgery. In a previous case, a patient underwent surgery to excise a mass which turned out to be a left atrial thrombus [13].

## Conclusions

Atrial thrombi should be suspected in patients with cardiomyopathy and symptoms of low flow states. Spontaneous contrast on echocardiography is highly correlated with left atrial thrombus on TEE. When present, a diagnosis of thrombus should be favored over myxoma. As there is a risk for thromboembolic events in both mural thrombi and myxomas, it is reasonable to offer anticoagulation to patients with low flow states even in the absence of atrial fibrillation while awaiting surgical evaluation. We suggest follow up echocardiography to confirm the persistence of intracardiac mass prior to surgical intervention to avoid needless surgery.
